# Epileptiform activity during inert gas euthanasia of mice

**DOI:** 10.1371/journal.pone.0195872

**Published:** 2018-04-19

**Authors:** Thomas C. Gent, Carlotta Detotto, Alexei L. Vyssotski, Regula Bettschart-Wolfensberger

**Affiliations:** 1 Anaesthesiology Section, Vetsuisse Faculty, University of Zürich, Zürich, Switzerland; 2 Institute for Neuroinformatics, ETH, Zürich, Switzerland; University of Bari, ITALY

## Abstract

Carbon dioxide (CO_2_) is one of the most commonly used euthanasia agents for mice, yet it is highly aversive and nociceptive. Inert gases are a possible alternative, however there are qualitative reports of seizures resulting from exposure. Here we evaluate epileptiform activity caused by inert gases (N_2_, He, Ar and Xe) and CO_2_ in mice chronically instrumented for EEG/EMG undergoing single-gas euthanasia. We found that N_2_, He and Ar caused epileptiform activity in all animals, CO_2_ in half of animals and no epileptiform activity produced by Xe. Atmospheric O_2_ concentrations at epileptiform activity onset were significantly higher for CO_2_ than for all other gases and occurred soon after loss of motion, whereas N_2_ and Ar epileptiform activity occurred at cessation of neocortical activity. Helium caused the longest epileptiform activity and these commenced significantly before isoelectric EEG. We did not detect any epileptiform activity during active behaviour. Taken together, these results demonstrate that whilst epileptiform activity from inert gases and particularly Ar and N_2_ are more prevalent than for CO_2_, their occurrence at the onset of an isoelectric EEG is unlikely to impact on the welfare of the animal. Epileptiform activity from these gases should not preclude them from further investigation as euthanasia agents. The genesis of epileptiform activity from CO_2_ is unlikely to result from hypoxia as with the inert gases. Helium caused epileptiform activity before cessation of neocortical activity and for a longer duration and is therefore less suitable as an alternative to CO_2_.

## Introduction

Carbon dioxide (CO_2_) is one of the most commonly used euthanasia agents for laboratory rodents, however its use is fraught with welfare concerns including fear, nociception and aversion [1–3]. There is an ongoing effort to find alternatives for which inert gases have been proposed as potential agents [4]. Inert gases are colourless odourless and non-irritant, which renders them attractive since they may be less aversive than CO_2_. Indeed, the use of nitrogen as a euthanasia agent for rats has been demonstrated not to cause an increase in heart rate or blood pressure, suggesting that the stress is lower than that experienced during CO_2_ euthanasia [5]. Furthermore, the potential for environmental pollution is lower and should be safer for human operatives performing the euthanasia.

Recent reports of argon gas euthanasia have raised significant concerns due to qualitative reports of seizure-like activity [6] and hyperreflexia [5] in rats. However, seizure-like activity has also been reported in rats undergoing CO_2_ euthanasia [2]. Crucially, the exact nature of this activity and the extent to which it impacts on the welfare of animals remains unknown. In this investigation, we used electroencephalography (EEG) and electromyography (EMG) combined with visual behavioural scoring, to determine the time course and behaviour of epileptiform activity caused by argon, nitrogen, helium, xenon and carbon dioxide in a mouse euthanasia paradigm.

## Methods

### Animals

We used adult male (8–12 weeks old, 25-30g) C57Bl6 mice (Charles Rivers Laboratories, Germany), chronically instrumented with EEG and EMG recording electrodes. Animals were kept in IVC cages on a 12:12hr light cycle and given access to standard laboratory rodent food and water *ad libitum*. All experiments were performed during the light period.

### Instrumentation

Animals were anaesthetised in isoflurane in oxygen and positioned in a stereotaxic frame, as previously reported [7]. Buprenorphine (100μg/kg), meloxicam (5mg/kg) and 0.9% saline (10ml/kg) were administered subcutaneously. The hair was then shaved from the scalp and the skin aseptically prepared. Holes were drilled in the skull and three small jewellery screws inserted above the dura (not penetrating brain tissue) to measure EEG. With respect to the cranial bregma suture, the ground electrode was placed +4.0 mm anterior and +1.0 mm lateral and the two recording electrodes -2.0 mm posterior and ±2.0 mm lateral. The recorded signal was a differential voltage between the two posteriorly placed electrodes. The bare ends of two wires were implanted in the rhomboideus muscles of the neck to measure EMG. All electrodes were then soldered to a pin connector and the implant sealed using methyl-methacrylate cement.

Animals were allowed two days to recover and were then habituated to wearing the Neurologger 2A recording device (see below) for 15 minutes each day for seven days. Experimentation was performed in the 9^th^ day after surgery.

### Experimentation/Recording

**Fig 1:**
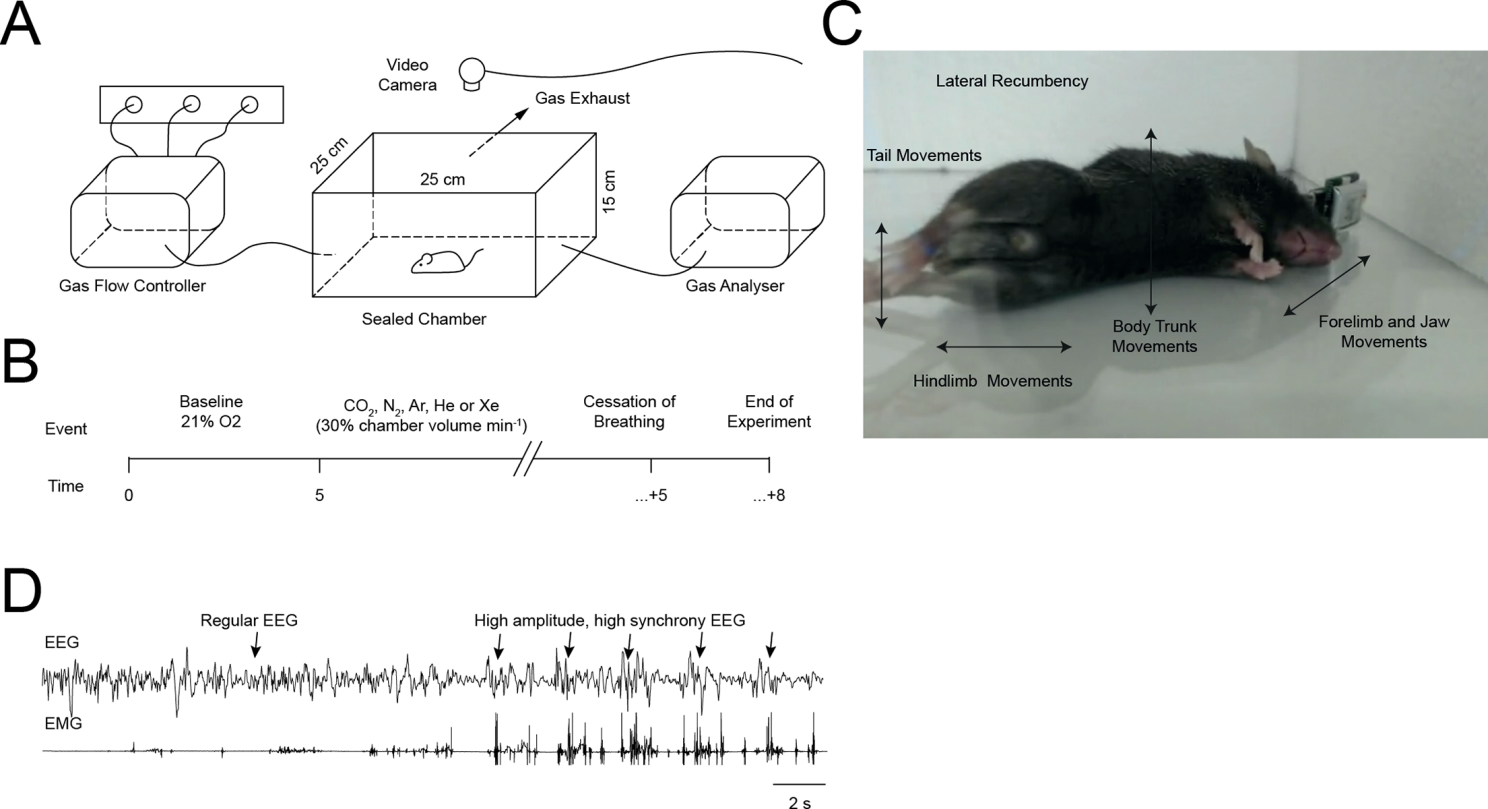
Experimental design. (A) Graphic representation of the experimental apparatus. The gas flow controller was calibrated to deliver precise amounts of each gas used and to switch from 21% oxygen at the end of the baseline period. (B) Timeline of the experimental procedure. (C) Graphic demonstrating the typical visual appearance of epileptiform activity. Not all elements were omnipresent, however lateral recumbency and hind limb movements were exhibited by all animals during epileptiform activity. (D) Example of electrophysiological appearance of an epileptiform event, taken from an Ar recording. Note the low muscle tone before onset. Epileptiform activity was characterised by high amplitude, highly synchronous bursting in the EEG. Note in this example the bursts are interspersed with very low EEG activity as the animal approaches cessation of neocortical activity.

Animals were randomised into one of five treatment groups, CO_2_, N_2_, He, Ar or Xe (n = 6 animals per group). Animals were connected to the Neurologger 2A [8] recording device and then returned to the home cage for 30 minutes. Individual animals were then transferred to a sealed chamber (length: 25cm, width: 25cm, height: 15cm; volume: 9.375 litres; Fig 1A) and a baseline in 21% oxygen recorded for 5 minutes. Gas was then infused into the chamber at 30% chamber volume per minute according to best practice guidelines [9], using a calibrated gas mixer (GSM-3, CWE Inc.). Air from the chamber was continuously sampled at a height of 3 cm from the chamber floor, via a 20 cm tube with an internal diameter of 6 mm at a rate of 1 L per minute. Oxygen concentration was measured at 1 Hz by a calibrated oxygen analyser (Rapidox 3100EA, Cambridge Sensotec) and recorded digitally. The experiment was terminated 3 minutes after cessation of breathing. Electrophysiological data was sampled at 200Hz with a low cut-off (3dB) filter of 0.5Hz. At the end of experimentation, data was downloaded from the Neurologger and analysed in Spike2 (CED, England).

### Epileptiform activity detection

Epileptiform activity was detected using retrospective analysis of video recordings of experiments, denoted by physical appearance of exaggerated and uncoordinated muscle activity (Fig 1B and 1C). Occurrence of epileptiform activity was scored if any one of the following criteria were noted during lateral recumbency: tail movements, hindlimb movements, body trunk movements, head and forelimb movements. Epileptiform activity was confirmed by simultaneous high amplitude, highly synchronous EEG activity with corresponding EMG activity (Fig 1D). Minimum thresholds of twice the signal amplitude of the previous two seconds of signal, were set for epileptiform event classification. Cessation of neocortical activity was determined as the point of consolidated isoelectric activity in the EEG. Loss of motion (LOM) was defined as the period when animals ceased any purposeful movements (with the exception of breathing) and were recumbent.

### Statistical analysis

Groups of data were analysed by one-way Anova with post-hoc Tukey’s modification with p-values less than 0.05 considered significant. Data was checked for normal distribution using Shapiro-Wilks test. Values in the text are reported as mean ± sem.

### Ethical approval

This work was approved by the Canton of Zürich veterinary office. License number: 58/2014.

## Results

### Epileptiform event prevalence

**Fig 2:**
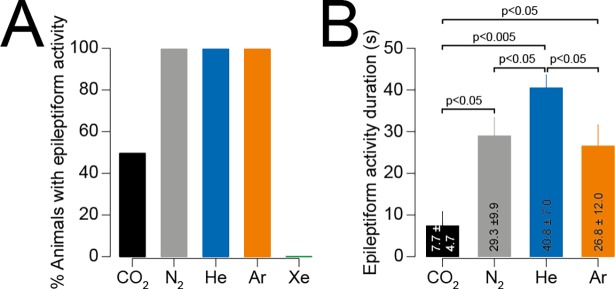
Demographics of epileptiform activity. (A) Prevalence of epileptiform events resulting from exposure to each gas. Note that electrophysiological epileptiform activity was exhibited by CO_2_; however, only one out of six animals demonstrated physical signs of epileptiform activity. Only Xe did not result in any epileptiform activity. (B) Duration of epileptiform events. CO_2_ produced the shortest epileptiform events, and He the longest. There was no difference between N_2_ and Ar.

Epileptiform activity resulting from CO_2_ exposure is not commonly reported. Therefore, we first determined the prevalence of epileptiform events caused by each gas. Epileptiform activity was found to occur in 100% of animals exposed to N_2_, He and Ar by both visual and EEG assessment whereas no epileptiform activity was found in any animal exposed to Xe (Fig 2A, S1 Fig and Table 1). Interestingly, we found that one out of the six mice exposed to CO_2_ had visual evidence of an epileptiform event, however a further two had epileptiform activity in the EEG that was not evident by visual scoring. Mice exposed to CO_2_ which did not exhibit epileptiform activity were excluded from further analysis. In all cases where visual evidence of epileptiform activity was found, there was corresponding epileptiform activity in the EEG.

**Table 1:** Experimental data and statistics.

	CO_2_	N_2_	He	Ar	Xe
**Prevalence (%)**	50	100	100	100	0
**Epileptiform event duration (s)**	7.7 ± 4.7	29.3 ± 9.9	40.8 ± 7.0	26.8 ± 12.0	-
**Time after LOM (s)**	13.7 ± 7.6	66.8 ± 12.8	30.4 ± 2.3	58.3 ± 12.4	-
**Time before cessation of neocortical activity (s)**	82.0 ± 19.1	1.3 ± 12.9	35.4 ± 12.8	2.0 ± 8.6	-
**Oxygen at LOM (%)**	14.0 ± 0.3	4.6 ± 0.2	3.9 ± 0.1	4.5 ± 0.1	9.6 ± 0.3
**Oxygen at epileptiform event onset (%)**	10.4 ± 0.4	4.9 ± 0.8	3.9 ± 0.2	4.8 ± 0.3	-

Data presented as mean ± S.E.M.

### Epileptiform event duration

Epileptiform events resulting from different physiological processes are likely to have differing durations. Therefore, we measured epileptiform event duration determined by the EEG (Fig 2B). We found that CO_2_ resulted in the shortest epileptiform activity (7.7 ± 4.7s, p < 0.05, n = 3 mice) whereas He produced the longest (40.8 ± 7.0, p < 0.05). There was no difference in epileptiform event duration for N_2_ and Ar (29.3 ± 9.9 vs 26.8 ± 12.0, p > 0.05) (Fig 2B and Table 1).

### Timing

**Fig 3:**
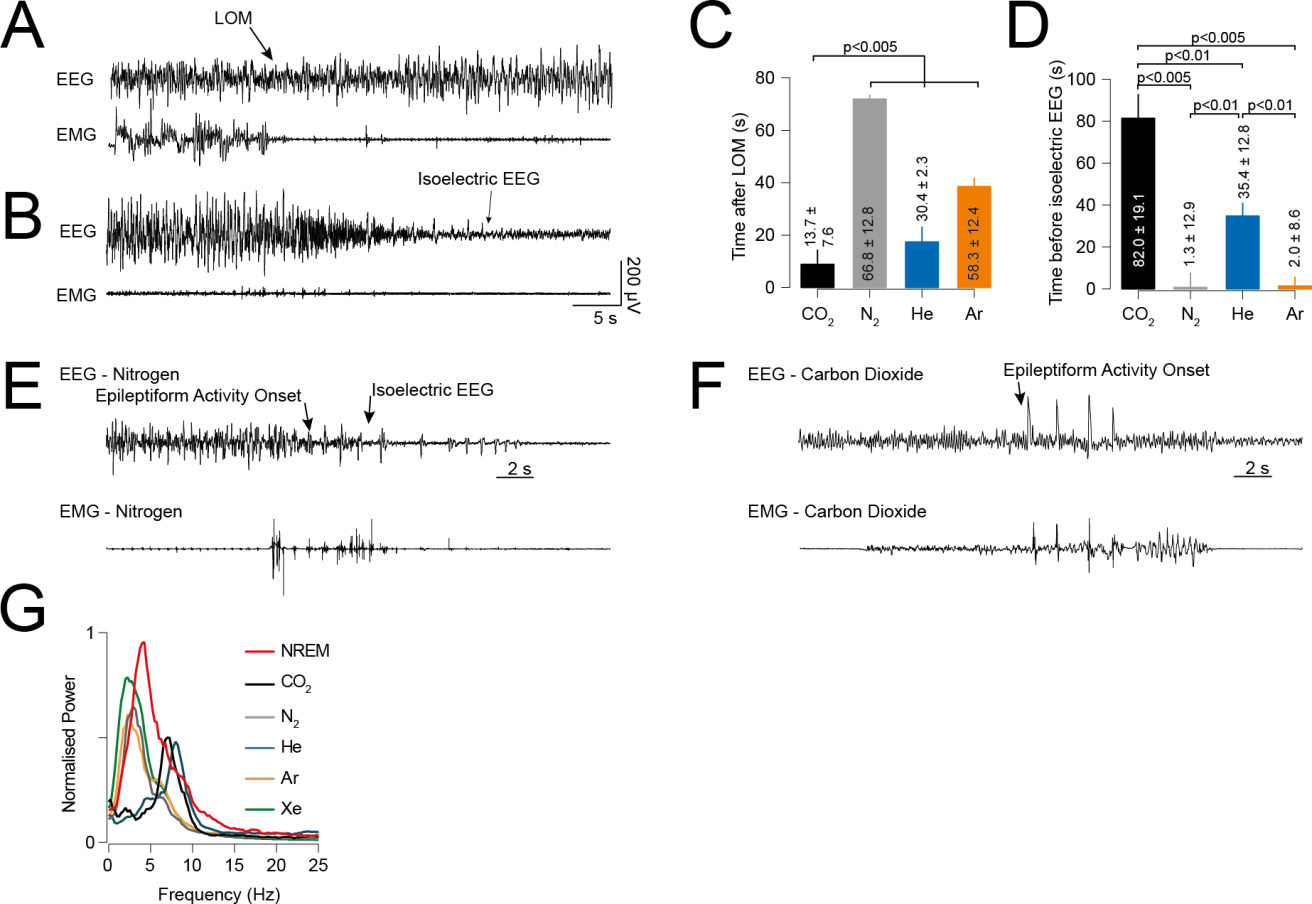
Timing of epileptiform event onset. (A) Representative EEG/EMG trace showing the criteria for determining LOM. Note the change in EEG from a low amplitude fast (awake) pattern to high amplitude slower rhythm. Note also that changes in EMG activity (LOM) occur several seconds before EEG activity changes. (B) Representative example from Xe recording (i.e.: no epileptiform activity) of the criteria for cessation of neocortical activity: defined as the point of onset of consolidated isoelectric EEG. (C) Time delay from LOM to epileptiform activity onset. Epileptiform events from CO_2_ occurred soon after cessation of neocortical activity. There was a significant delay for other gases, with He induced epileptiform activity occurring latest. (D) Time of epileptiform activity onset before cessation of neocortical activity. N_2_ and Ar epileptiform activity occurred at the point of cessation of neocortical activity. (E), (F) Representative EEG/EMG traces of the onset of epileptiform activity and cessation of neocortical activity for N_2_ and CO_2_ respectively. Note the EMG tone occurring after cessation of neocortical activity, which indicates the continued activity of spinal and brainstem reflexes. (G) Normalised power spectra of EEG for 15s after LOM and also natural sleep (NREM). CO_2_ and He resulted in brain activation whereas other gases reduced cortical arousal compared to sleep.

The temporal relationship of epileptiform event onset to loss of motion (LOM) and cessation of neocortical activity is likely to determine perception of the event by the animal. To compare the gases used, we measured the time of onset of the epileptiform event after LOM and before cessation of neocortical activity (Fig 3A and 3B). We found that the onset of CO_2_ epileptiform events occurred rapidly after LOM (13.7 ± 7.6s, p < 0.005) whereas epileptiform activity onset was significantly delayed for other gases (Fig 3C and Table 1). Furthermore, we found that N_2_ and Ar epileptiform events occurred at the point of cessation of neocortical activity (1.3 ± 12.9s vs. 2.0 ± 8.6s). However, epileptiform events induced by CO_2_ (82.0 ± 19.1) and He (35.4 ± 12.8) occurred significantly before cessation of neocortical activity (Fig 3D, 3E and 3F and Table 1). All detected epileptiform events occurred following LOM as determined by video tracking and was associated with a predominating large-amplitude activity in the EEG and low EMG tone. Furthermore, we found that this activity differed in nature between gases (Fig 3G and Table 1). We compared the normalised power spectra of the EEG for the first 15s after LOM and also that of natural sleep (NREM); as many of the animals fell asleep in the home cage prior to being transferred to the chamber. We found that none of the EEG signatures matched that of natural sleep. For Xe, N_2_ and Ar, the EEG was dominated with a lower frequency power, similar to that induced by many general anaesthetics [10]. Interestingly, He and CO_2_ EEGs were mainly faster lower amplitude rhythms indicating neocortical activation.

### Effect of hypoxia

**Fig 4:**
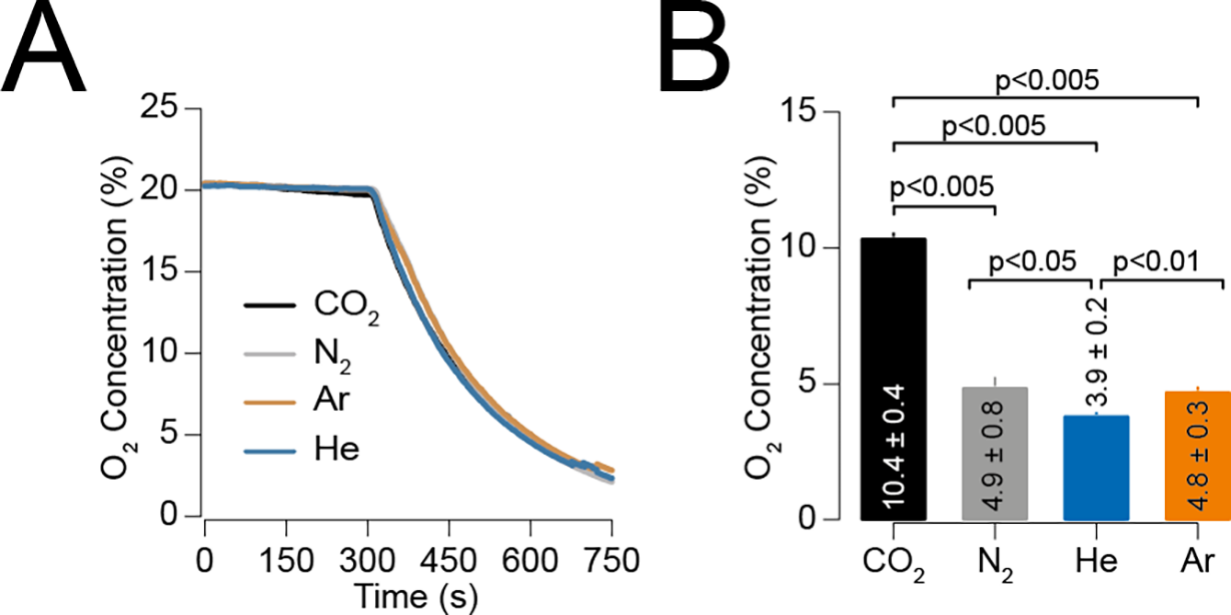
Oxygen concentration at epileptiform event onset. (A) Average oxygen titration curves during gas recordings, starting with a 5-minute baseline at 21% oxygen. There was no difference between groups. (B) Oxygen concentrations at epileptiform event onset. CO_2_ epileptiform events started at higher oxygen concentrations than other gases. Note that He epileptiform activity started at lower oxygen concentrations than N_2_ and Ar.

CO_2_ has true narcotic properties, whereas loss of consciousness from exposure to inert gases is most likely to occur due to hypoxia (with the exception of Xe which is a general anaesthetic). However, since He produced epileptiform events that were different in duration and onset compared to Ar and N_2_, we measured the oxygen concentration in the chamber at the point of epileptiform event onset to determine the role of hypoxia. The titrations in oxygen concentration were the same for all groups and therefore epileptiform event onset was not time-dependant (Fig 4A). Furthermore, there was no difference in the oxygen concentration at LOM for Ar, N_2_ and He (p > 0.05; Table 1).

We further found that CO_2_ epileptiform events occurred at significantly higher O_2_ concentrations than the inert gases (10.4 ± 0.4%, p < 0.005). Both N_2_ and Ar induced epileptiform activity occurred at the same concentration (4.9 ± 0.8% vs. 4.8 ± 0.3%) whereas He epileptiform events started at significantly lower concentrations (3.9 ± 0.2%, p < 0.05, Fig 4B and Table 1).

## Discussion

The practice of using CO_2_ for euthanasia of laboratory rodents is highly speculative, however suitable alternatives are yet to be found [4]. One of the major reservations against inert gas euthanasia is that of seizures which was described for Ar [6], however other inert gases are yet to be thoroughly investigated. Here, we demonstrated inert gas euthanasia produces epileptiform events rather than ongoing seizure activity, as shown in EEG traces. We further found that CO_2_ euthanasia does produce epileptiform activity and whilst they are shorter in duration and apparently less severe than those resulting from hypoxia, the incidence of CO_2_ epileptiform events may be underreported since they are not always visible. Furthermore, the epileptiform activity genesis is likely to have a different mechanism than those from inert gas exposure, since they occur at oxygen concentrations that are significantly higher. This mechanism is not currently understood, although CO_2_ exposure at this level results in severe acidosis [11] and increases in intracranial pressure [12], both of which may trigger epileptiform events. Most interestingly is that Xe exposure does not result in epileptiform activity despite the fact that cessation of neocortical activity occurs at oxygen concentrations that are even lower than those of the other inert gases [13]. This concurs with observations in rodents [14] and humans [15]. The reasons for this are not entirely clear, however it is highly likely that a combination of neuroprotection [16] and preservation of cardiac function [17] result in the brain maintaining sufficient oxygenation to offset any epileptiform activity. Additionally, Xe has true hypnotic properties that reduce neuronal excitability and will raise the epileptiform activity threshold [15], unlike hypoxia which increases excitability before cell death occurs [18]. CO_2_ also has hypnotic properties, however following LOM it caused neocortical activation, unlike N_2_, Ar and Xe. Helium also resulted in neocortical activation which may explain the prolonged epileptiform activity that it caused.

It would seem reasonable to assume that all other inert gases would result in a purely hypoxic death and would therefore result in epileptiform activity that was similar and predictable. However, we found that epileptiform activity resulting from He exposure differed significantly from N_2_ and Ar. Interestingly, He epileptiform events started at more hypoxic levels than N_2_ and Ar and persisted longer, however paradoxically occurred longer before cessation of neocortical activity. The reasons for this are also unclear however He is neuroprotective but non-anaesthetic [19]. It is possible that its neuroprotective effects offset neuronal excitability to more extreme levels of hypoxia, but are unable to completely prevent them since it lacks the membrane stabilising properties of Xe [20]. Furthermore, the increased EEG frequency during He was similar to CO_2_, not the other inert gases. Such activity is typically associated with increased neuronal activity compared to the slower rhythms of NREM [7] and might therefore predispose to epileptiform activity.

Whilst unintentional epileptiform activity in laboratory rodents is clearly undesirable for any intervention, consideration should be given to the perception of the epileptiform event by the animal to determine its welfare implications. We found that epileptiform activity from N_2_ and Ar exposure occurred at the point of cessation of neocortical activity where the mice had most likely been unconscious for some time. This would suggest that such motor movements were under subcortical and spinal control only [21]. Hyperreflexia from Ar exposure was reported at the onset of unconsciousness in rats [5], however we did not note any such activity until much later. For CO_2_ epileptiform events however, the onset was much sooner after LOM. A recent working group concluded that following the onset of unconsciousness, welfare concerns of euthanasia techniques ceased [4]. Conscious perception is defined as physiological response to a stimulus [22]. We used LOM as a surrogate for loss of consciousness [23] since we hypothesised that mice would remain active in a novel environment for a sustained period. In the short term, it is possible that muscle weakness from hypoxia would result in loss of motion before loss of consciousness, particularly since we observed a change in EMG tone prior to significant changes in EEG oscillations (Fig 3A). However, are unable to conclude at which point consciousness was lost at the same time as we did not measure evoked potentials in these experiments and therefore are unable to determine from these results whether any of the epileptiform events resulted in ‘suffering’. It is feasible that epileptiform activity occurring soon after LOM (such as those exhibited by CO_2_ exposure) may result in some consciousness perception whereas epileptiform activity which occurs at the point of cessation of neocortical activity is extremely unlikely to result in any perception. This requires further experimental verification.

Collectively these findings would suggest that whilst epileptiform activity prevalence from euthanasia of mice exposed to N_2_ and Ar are high, the nature of the epileptiform events make them unlikely to pose a real impingement on animal welfare. However, selection of an ideal euthanasia agent including factors such as aversion, fear and nociception should also be considered, were not objectives of our study. We would therefore argue that the previously documented epileptiform activity from Ar exposure [6], should not *per se* preclude it from further investigation as an alternative to CO_2_.

## Supporting information

S1 Fig Raw traces of epileptiform activity. Twenty second traces from all animals in each group at periods showing epileptiform activity, or time matched periods when no epileptiform activity was exhibited (CO_2_ and Xe). Epileptiform activity periods are highlighted by red boxes.
(TIF)
