# Supplementary figures and images for: Epileptiform activity during inert gas euthanasia of mice
Source: PLoS One. 2018 Apr 19;13(4):e0195872. doi: 10.1371/journal.pone.0195872 (PMC5908136; doi:10.1371/journal.pone.0195872)

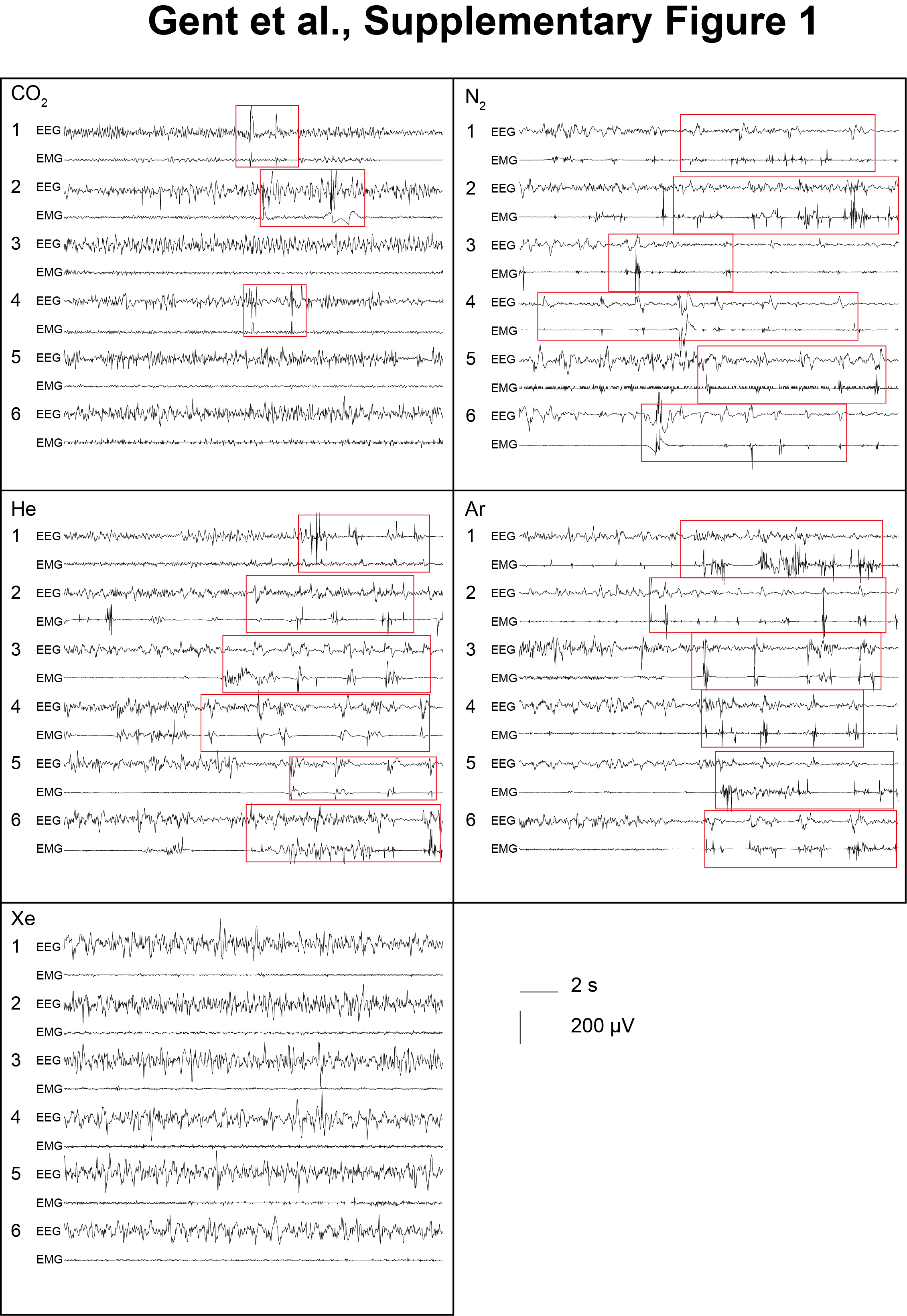

Supplement: S1 Fig — Twenty second traces from all animals in each group at periods showing epileptiform activity, or time matched periods when no epileptiform activity was exhibited (CO2 and Xe). Epileptiform activity periods are highlighted by red boxes. (TIF) [file pone.0195872.s001.tif]
